# Delayed Awareness of the History of Barium Examination: Perforated Barium Appendicitis

**DOI:** 10.1155/2017/6316175

**Published:** 2017-04-09

**Authors:** Susumu Saigusa, Masaki Ohi, Satoshi Oki, Takashi Ichikawa, Minako Kobayashi, Yasuhiro Inoue, Chikao Miki

**Affiliations:** ^1^Department of Surgery, Iga City General Hospital, 831 Shijuku-cho, Iga, Mie 518-0823, Japan; ^2^Department of Gastrointestinal and Pediatric Surgery, Mie University Hospital, 2-174 Edobashi, Tsu, Mie 514-8507, Japan; ^3^Department of Surgery, Wakaba Hospital, 28-13 Minami-Chuo, Tsu, Mie 514-0832, Japan

## Abstract

A 41-year-old man presented to our hospital with lower abdominal pain and a high-grade fever. Physical examination revealed rebound tenderness and guarding in the lower abdomen. Abdominal X-ray examination showed a radiopaque object in the right lower quadrant of the abdomen. Abdominal computed tomography (CT) demonstrated that the object had a strong artifact with over 10,000 Hounsfield units, as well as ascites around the terminal ileum. We diagnosed acute peritonitis with a suspicion of the perforation due to unknown foreign body and performed an emergency laparotomy. Operative findings showed a contained perforation of a phlegmonous appendicitis, and appendectomy was performed. The resected specimen demonstrated that the appendix contained a fecalith, and histopathological examination showed the crystal structure of barium sulfate in the lumen of the appendix. Unfortunately, we did not obtain the history of screening for gastric cancer using a barium examination one month prior to our appendectomy. Our experience demonstrates the importance of establishing a history of barium examinations of the gastrointestinal tract in a patient with a radiopaque object in the right lower quadrant of the abdomen for early diagnosis of barium appendicitis. Additionally, early diagnosis of barium appendicitis may affect the selection of surgical procedures.

## 1. Introduction

Barium appendicitis is a rare clinical entity and a known complication of barium examinations [1, 2]. The pathogenesis of barium appendicitis is unclear, however, and several mechanisms have been reported [3]. Radiographic differential diagnoses for a radiopaque object in the right lower quadrant of the abdomen include right ureteral lithiasis, colonic diverticulum with a calcified fecalith, and a foreign body. The shape and radiopaque density of an object on imaging is misleading and may not suggest barium appendicitis [4–6]. Therefore, comprehensive examination including medical history, physical examination, laboratory findings, and radiological imaging is required for the early diagnosis of barium appendicitis. We report a case wherein preoperative diagnosis of barium appendicitis was difficult due to the shape, location, and strength of the artifact of the object on CT.

## 2. Case Presentation

**Figure 1:**
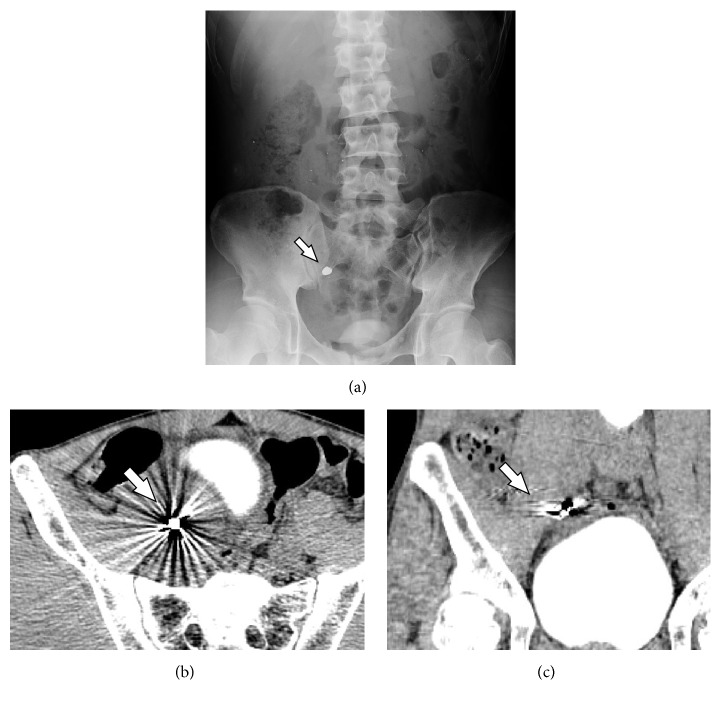
Abdominal X-ray examination. Radiopaque object consistent with a metallic foreign body in the right lower quadrant of the abdomen (a). Retained barium with strong artifact on axial (b) and coronal (c) views on abdominal CT.

**Figure 2:**
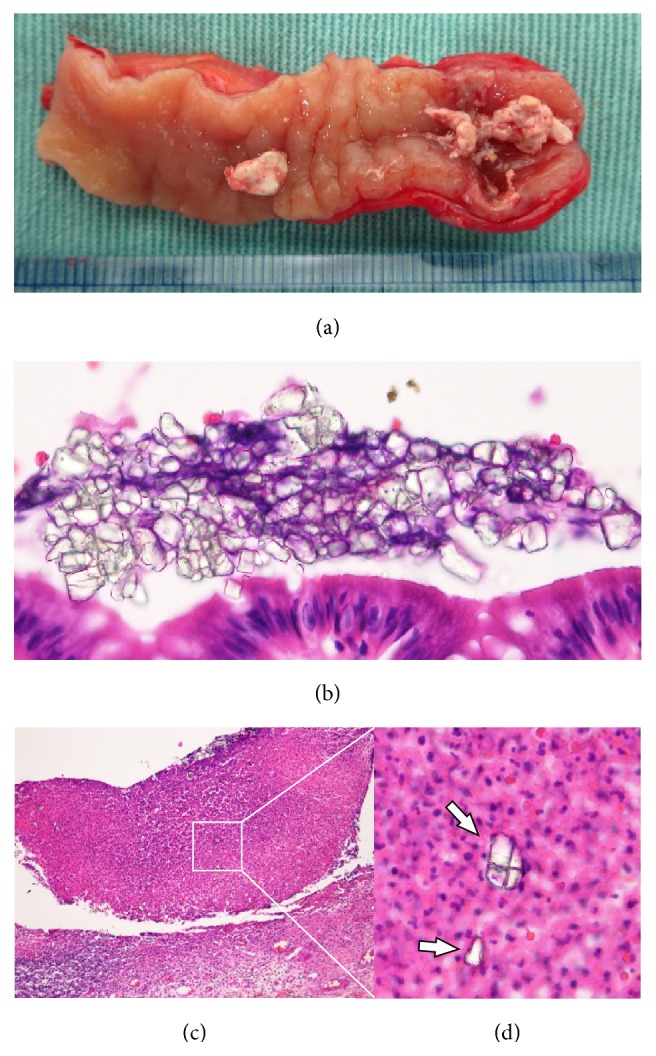
Resected specimen shows that the appendix contained a white, fragile fecalith. There was inflammation at the tip of the appendix (a). Crystal structure of barium sulfate on the mucosa (b) and in the infected fluid (c, d). Original magnification 200x (c), 400x (b, d).

A 41-year-old man presented to our hospital with lower abdominal pain and a high-grade fever. Physical examination revealed rebound tenderness and guarding in the lower abdomen. Abdominal X-ray examination showed a radiopaque object suggestive of a foreign body in the right lower quadrant of the abdomen (Figure 1(a)). Abdominal contrast-enhanced computed tomography (CT) showed that the object had strong artifact with more than 10,000 Hounsfield units, as well as ascites around a thickened terminal ileum (Figures 1(b) and 1(c)). On laboratory investigation, C reactive protein (CRP) level was elevated at 13.55 mg/dl (normal range < 0.45 mg/dl), and white blood cell count was 9900/*μ*l. We performed an emergency laparotomy given the patient's evidence of peritonitis on physical exam. Operative findings showed a contained perforation of a phlegmonous appendicitis with purulent ascites, and appendectomy was performed. Intraoperative X-ray confirmed that the object was removed after appendectomy. The resected specimen had within it a fragile, white, fecalith (Figure 2(a)). Ascites cultures showed* Pseudomonas aeruginosa*. Histopathological examination showed ulceration, necrosis, and an abscess in the mucosal layer of the appendix and the crystal structure of barium sulfate in the lumen and within the abscess (Figures 2(b)–2(d)). We administered meropenem (1 g/day) from admission until the third postoperative day. Postoperatively, it was revealed to us that he had undergone screening for gastric cancer using barium one month earlier. The postoperative course was uneventful.

## 3. Discussion

Radiological findings of retained barium are variable [1], and they are frequently confused with metal foreign bodies. Adachi et al. reported an object in the right lower quadrant of the abdomen and, based on the shape and radio-density, led to a preoperative diagnosis of a localized intra-abdominal abscess due to ileocecal perforation secondary to a metallic foreign body such as a dental crown [5]. Similarly, we strongly suspected a foreign metal body in the appendix as the abdominal CT showed that the object had a strong artifact with over 10,000 Hounsfield units. Coronal and sagittal views on CT may aid in diagnosis of barium appendicitis, but differentiation of barium from a metallic object remains difficult. Therefore, a detailed medical history, physical examination, laboratory studies, and radiological imaging are required for early diagnosis of barium appendicitis. In particular, a history of gastrointestinal examination using barium is important. Li et al. reported that barium examination of the gastrointestinal tract has a time-dependent association with appendicitis risk [7].

Barium sulfate is widely used for gastrointestinal imaging and harmful complications are rare. Barium is usually excreted by patients within 3 days after gastrointestinal imaging [3]. However, there are several reports in the literature describing cases of barium appendicitis, with little known about the pathophysiology [2, 3, 8]. The interval before the development of barium appendicitis has been reported to range from a few hours to a few months [1]. Maglinte et al. reported on 31 patients with retained barium in the appendix for longer than 72 hours and followed them for 1 year. They failed to find any association between barium retention and the development of acute appendicitis [9]. This suggests that not all retained barium leads to appendicitis. Therefore, prophylactic appendectomy for retained barium in the appendix is not recommended [6]. On the other hand, when barium appendicitis is definitively diagnosed, appendectomy should be immediately performed as barium appendicitis can lead to perforation [1].

Several authors reported the comparison of clinical outcome between open and laparoscopic appendectomy, and they concluded that laparoscopic appendectomy for acute appendicitis with or without its perforation had beneficial advantages including cost-effectiveness and decrease of wound infection [10–12]. However, we selected open appendectomy but not laparoscopic approach in the present case because we preoperatively could not diagnose barium appendicitis with peritonitis. If preoperative diagnosis of barium appendicitis was given, laparoscopic approach could be considered as an option for the treatment. Hence, early diagnosis of barium appendicitis may be important to make choice of surgical approach.

In conclusion, our experience indicates that barium appendicitis should be taken into consideration for patients with a radiopaque object in right lower quadrant abdomen. Emergency physicians especially should be aware of and alert to possibility of barium appendicitis when facing similar clinical course. Additionally, early diagnosis of barium appendicitis may be important for selection of surgical procedure.
